# New Phenolic Compounds in *Posidonia oceanica* Seagrass: A Comprehensive Array Using High Resolution Mass Spectrometry

**DOI:** 10.3390/plants10050864

**Published:** 2021-04-25

**Authors:** Marina Astudillo-Pascual, Irene Domínguez, Pedro A. Aguilera, Antonia Garrido Frenich

**Affiliations:** 1 Department of Biology and Geology, International Campus of Excellence in Marine Science (CEIMAR), University of Almeria, E-04120 Almeria, Spain; m.astudillo@ual.es (M.A.-P.); aguilera@ual.es (P.A.A.); 2 Department of Chemistry and Physics, Research Centre for Mediterranean Intensive Agrosystems and Agri-Food Biotechnology (CIAIMBITAL), Agrifood Campus of International Excellence ceiA3, University of Almería, E-04120 Almeria, Spain; agarrido@ual.es

**Keywords:** *Posidonia oceanica*, leaf, rhizome, root, UHPLC-Orbitrap MS, phenolic compounds

## Abstract

The studies on the *Posidonia oceanica* Delile (*P. oceanica*) phenolic composition have been focused on the foliar tissues and have often neglected the phenolic compounds in rhizomes or roots alike. With the current improvements in high resolution mass spectrometry (HRMS) analyzers, such as the Orbitrap MS, there is a new opportunity to more deeply study *P. oceanica*. One of the benefits is the possibility of conducting an exhaustive phenolic monitoring, which is crucial in the search for new stressor-specific biomarkers of coastal deterioration. For this purpose, the different tissues (leaf, rhizome, and root) of *P. oceanica* seagrass from several marine sampling areas were analyzed through target, suspected, and non-target screenings. This paper brings a fast and tissues-specific extraction, as well as a detection method of phenolic compounds applying for the first time the potential of HRMS (Exactive Orbitrap) in *P. oceanica* samples. As a result, 42 phenolic compounds were satisfactorily detected, of which, to our knowledge, 24 were not previously reported in *P. oceanica*, such as naringenin, naringenin chalcone and pinocembrin, among others. Information here reported could be used for the evaluation of new stressor-specific biomarkers of coastal deterioration in the Mediterranean waters. Furthermore, the followed extraction and analytical method could be considered as a reference protocol in other studies on marine seagrasses due to the exhaustive search and satisfactory results.

## 1. Introduction

*Posidonia oceanica* Delile (*P. oceanica*) is a long-living, slow-growing marine angiosperm (seagrass) endemic to the Mediterranean Sea. The *P. oceanica* meadows have been identified as priority habitats by European conservation legislation [1]. These meadows supply essential ecosystem services for human well-being [2]. However, *P. oceanica* has been observed to be especially susceptible to anthropogenic disturbances [3,4,5]. Studies have detected the presence and bioaccumulation of pollutants in this seagrass, such as heavy metals. Ref. [6] brought insight into their application as contamination bioindicators. It has also been reported that *P. oceanica* responds to contamination-driven stress as well as to natural-driven stress (grazing, competition, microbial settlement, and eutrophication among others) by altering the amount of phenolic compounds [7,8]. Due to their specific and rapid response when changes in the environment occur, phenolic compounds have been proved to be suitable biomarkers or early warnings [9,10,11,12]. These compounds are specialized metabolites that benefit the bearing organism through several biological properties (e.g., antioxidant capacity) [13]. Interest in this field has been growing, given the potential medical contributions of such phenolic compounds to human health and personal care: immunostimulant and antitumoral drugs or products for skin aging [14,15].

*P. oceanica* phenolic content has been previously reported [14,16]. Of the researched compounds, ferulic acid, caffeic acid, chicoric acid, caftaric acid, and cinnamic acid have been identified as major phenolic compounds [17,18]. Studies commonly focused on the determination of phenolic compounds in foliar tissues (temporal structures marked by seasonal variations), and little attention has been paid to the tissues with a longer lifespan less marked by seasonal variations, i.e., rhizomes and roots. To the best of our knowledge, only one study reported the phenolic fingerprint in *P. oceanica* roots [19], and available studies based on rhizomes have only reported total phenols (following mainly the Folin–Ciocalteu assay) or lignin content [18,20,21,22]. However, several studies on terrestrial plants have observed specific phenols in these tissues reacting to specific changes in the environment or taking part in relevant activities, such as the reduction of contaminants in soil or their role in plant resistance to deficit conditions [23,24,25].

Additionally, studies based their identification/detection of the phenolic compounds on liquid (LC), gas chromatography (GC), or pyrolysis GC (Py-GC) coupled to low resolution mass spectrometry (LRMS) [15,18,26]. Nowadays, the improvements in high resolution mass spectrometry (HRMS) and its ability to detect a higher number of compounds, bring a new opportunity to obtain a comprehensive array of phenolic compounds in *P. oceanica*. The implementation of HRMS analyzers could also lead to (1) a better differentiation between genuine signals and artifacts driven signals, (2) accurate detection of target and non-target compounds, and (3) retrospective analysis of samples.

Hence, the objective of this study is double fold. Firstly, the determination of phenolic compounds in *P. oceanica* for the first time, to our knowledge, by ultra-high performance liquid chromatography combined with HRMS, concretely with an Orbitrap analyzer (UHPLC-Orbitrap MS), using target, suspected, and non-target analysis. Secondly, the evaluation of the phenolic content in the different *P. oceanica* tissues (leaf, rhizome, and root) from different sampling points. The findings of this study provide new reference information in the field of marine seagrass chemistry that could be useful in future investigations of *P. oceanica* phenolic compounds’ suitability as stress-specific biomarkers.

## 2. Results

### 2.1. Extraction Procedure

**Figure 1:**
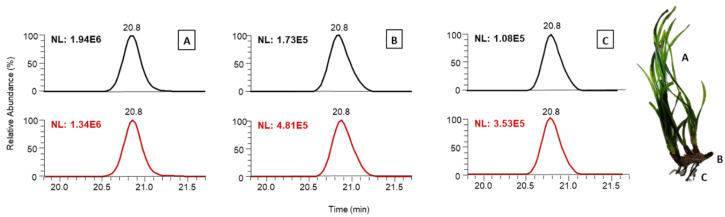
Extracted ion chromatogram for isorhamnetin-3-O-glucoside at 479.11840 *m/z* in full MS, with *R_T_* 20.8 min, for FAN7 tissues: (**A**) leaves, (**B**) rhizomes, and (**C**) roots showing differences in base peak intensities (NL). Black chromatographic peaks were obtained using 5:5 *v*/*v* extraction mixture and brown peaks correspond to 8:2 *v*/*v* extraction mixture.

Two different procedures were compared in the tissues of one of the collected *P. oceanica* samples (FAN7 leaves, rhizome, and roots) for extractant composition evaluation [17,27]. Procedures varied between a mixture of methanol/water 8:2 *v*/*v* and 5:5 *v*/*v* (pH 4 in both cases). After ca. 15 min extraction, samples were subject to a target screening using an in-house database. This database used 93 commercially available standards of the most common phenolic compounds detected in agri-food products [28,29]. In this preliminary screening, up to 20 compounds were identified and considered for the extractant composition evaluation. The results showed that the optimal composition was tissue-dependent (Table S1). For instance, for rhizomes and roots, an extractant mixture of 8:2 *v*/*v* methanol/water was proved to be the most adequate in terms of phenolic profile sensitivity in all cases, i.e., number of compounds, higher peak intensities (described as NL, Normalization Level), better peak shape, or a greater presence of confirmation fragments. In contrast, leaves showed overall relatively better results when using the 5:5 *v*/*v* mixture (~72% of the cases). An example of how the intensity varied according to the employed extractant composition and the studied tissue is shown in Figure 1. Observed differences in extraction efficiencies could be attributed to the type of plant material (e.g., ligneous and rigid in the case of the rhizome and roots) and the compounds affinity to the different tissues.

The results obtained for leaves confirmed that the extraction followed in previous studies (5:5 *v*/*v* mixture) yield relatively better results than the 8:2 *v*/*v* mixture [15,16,17,30]. Nonetheless, the scarcity of data on roots and rhizomes hindered the comparison between our results and other published procedures. No available information on rhizomes was found and, after a thorough search of the relevant literature, only one article was found regarding the extraction of the roots, in which non-diluted acetone was used in a three day extraction procedure [19]. In all observed articles, extraction times ranged from several hours to days or consumed elevated solvent-to-plant. In contrast, the extraction method employed in this study proved to be less time consuming and employed a reduced solvent volume, following the green chemistry approach.

Therefore, based on the assessment of the results, the extractant composition selected was a mixture of methanol/water 5:5 *v*/*v* for leaves, and 8:2 *v*/*v* in the case of rhizome and roots which allowed for the detection of a greater number of compounds (up to four) for each tissue.

### 2.2. Chromatographic Conditions

Two sub-2-μm columns, widely employed in the phenolic compound’s studies, were also tested in the analysis of FAN7 leaves. A target screening was performed using the in-house database to evaluate the chromatographic separation performance. Such columns were Acquity C18 column (2.1 mm × 100 mm, 1.7 μm particle size; Waters, Milford, MA, USA) and Hypersil GOLD^TM^ (2.1 mm × 100 mm, 1.9 μm particle size; Thermo Fisher, San Jose, CA, USA).

Besides, two common aqueous mobile phases were also evaluated, 30 mM ammonium acetate aqueous solution pH 5 [27] and 4 mM formic acid aqueous solution pH 3 [17]. Methanol was employed as the organic eluent along the process.

Regardless of the employed columns, compound separation remained the same. On the other hand, elution times differed, showing an increase in *R_T_* when using the Acquity C18 column, which could be attributed to the more reduced particle size. However, overall, relatively higher intensities or NL were reached when using Hypersil GOLD^TM^, as shown in Figure S1.

As for the mobile phases and their respective pH, they have been seen to affect the peaks’ intensity and the presence or absence of compounds and their confirmation fragments. In general, intensities were lower when ammonium acetate was employed, which in some cases hindered the apparition of confirmation fragments. Average better signals were observed in the formic acid mobile phase with pH 3, confirming effective binding of the phenolic compounds to the stationary phase. Up to six compounds more were observed when using formic acid. Examples are caffeic acid, p-coumaric acid or ferulic acid, phenolic compounds that were only confirmed when using formic acid. As an example, the case of ferulic acid is shown in Figure S2, which was absent when using ammonium. Such absence might be due to the charge of ferulic acid in increased pH, hampering the binding to the column.

As a result, the selected conditions for this analysis were: Hypersil GOLD^TM^ column, and formic acid as the aqueous mobile phase.

### 2.3. Phenolic Identification

After evaluating the extraction conditions, target, suspected, and non-target screenings of all tissues (leaves, rhizome, and roots) of five *P. oceanica* seagrasses were conducted as indicated in Figure S3. During the target screening, compound peaks of each molecule were located using information from the in-house database, such as the parent theoretical mass and confirmation/characteristic fragments, and performing pseudo MS/MS experiments. The risk of false-positive was also reduced by monitoring each parent’s peak mass spectrum and comparing it to the theoretical molecule spectrum (or simulation) to confirm the ion ratios for the isotopic pattern [28].

Results of the target screening revealed 22 phenolic compounds from which 13 were detected for the first time to our knowledge in *P. oceanica* (Table 1). These newly reported specialized metabolites consisted of flavones (apigenin, baicalein, and luteolin), flavonols (galangin), isoflavones (biochanin A, genistein, and glycitein), flavonons/flavanones (eriodictyol, naringenin, pinocembrin, and sakuranetin/isosakuranetin), chalcones (naringenin chalcone), and kaempferol-3-O-glucoside/luteolin-4’-O-glucoside (IUPAC names can be found in Table S2). These phenolic compounds have already been proved to play essential roles inhibiting cancer or offering antioxidant properties [15,31,32]. More biological properties are summarized in Table S3, Supplementary Materials.

Note that some of the detected phenolic compounds were isomers, with the same exact mass, *R_T_*, and fragments. Since they could not be separated by chromatographic or mass resolution and the presence of both isomers could not be confirmed, these were recorded and counted as only one phenolic compound [28]. These phytochemicals were: luteolin-4′-O-glucoside and kaempferol-3-O-glucoside (*m/z* 447.09328; *R_T_*: 20.7–20.8 min), and isosakuranetin and sakuranetin (*m/z* 285.07685, *R_T_*: 34.4 min).

**Figure 2:**
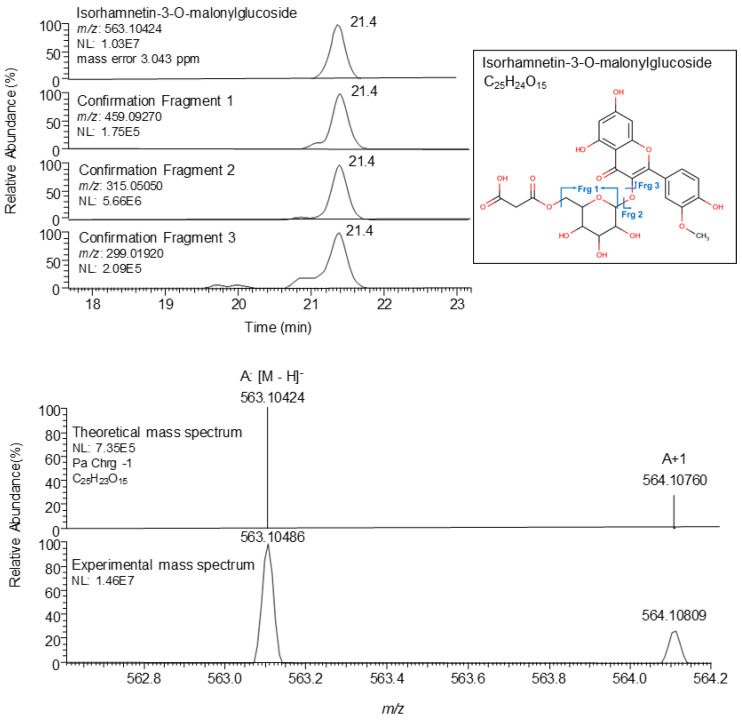
From top to bottom: Extracted ion chromatogram and mass spectrum of the suspected bioactive compound isorhamnetin-3-O-malonylglucoside (*m/z*: 563.10424) in foliar tissues from the sampling point AL2x. NL: intensity.

For the suspected screening, a second list with 38 compounds was developed by gathering published information on the *P. oceanica* phenolic fingerprint and other Mediterranean seagrasses (namely *Cymodocea nodosa*, *Zostera marina,* and *Zostera noltii*). Out of the 30 considered suspected compounds previously detected in *P. oceanica,* nine were tentatively identified in the samples and included in Table 2. On the other hand, the not detected suspected compounds are shown in Table S4. Note that the tentative identification of a compound was reached when (1) the difference between the exact mass of the candidate (calculated from the elemental formula) and the exact mass of the target compound felt within ±5 ppm of mass error, (2) confirmation fragments were present and, (3) spectrum matched among experimental and theoretical peaks in terms of ion ratios for the isotopic pattern (Table 2). Figure 2 shows a chromatogram and mass spectrum of isorhamnetin-3-O-malonylglucoside as an example. Suspected compounds identified in other Mediterranean seagrasses, such as apigenin-7-O-glucoside and rosmarinic acid or the sulfated flavonoids apigenin-7-sulfate, diosmetin-7-sulfate, and luteolin 7-sulfate, were not detected in this study in agreement with the literature [32,33,34].

During the non-target screening, the Compound Discoverer Software (Thermo Fisher Scientific, Les Ulis, France) was employed to identify potential candidates. In this search, the software subtracts all exact masses and the respective molecular formulas. Finally, these formulas were compared to information on phenolic compounds gathered in open databases, searching for matches. Only those highlighted as a full match and meeting the criteria settled for a tentative identification were considered. The non-target analysis revealed 11 compounds not previously identified in *P. oceanica*, such as sophoraflavanone B and two curcuminoids (Table 3 and Table S2). In addition, through the non-target screening several compounds detected during the target and suspected analysis were further confirmed: chicoric acid, p-coumaric acid, ferulic acid, fertaric acid, bioachin A, genistein, naringenin, isorhamnetin, isorhamnetin-3-glucoside, and quercetin-3-O-glucoside. The fact that certain compounds were only observed in the non-target screening whilst others were only detectable during the target and suspected mode highlights the need to combine the three searching modes and the benefits of the retrospective analyses for environmental samples, as recently suggested [36].

**Figure 3:**
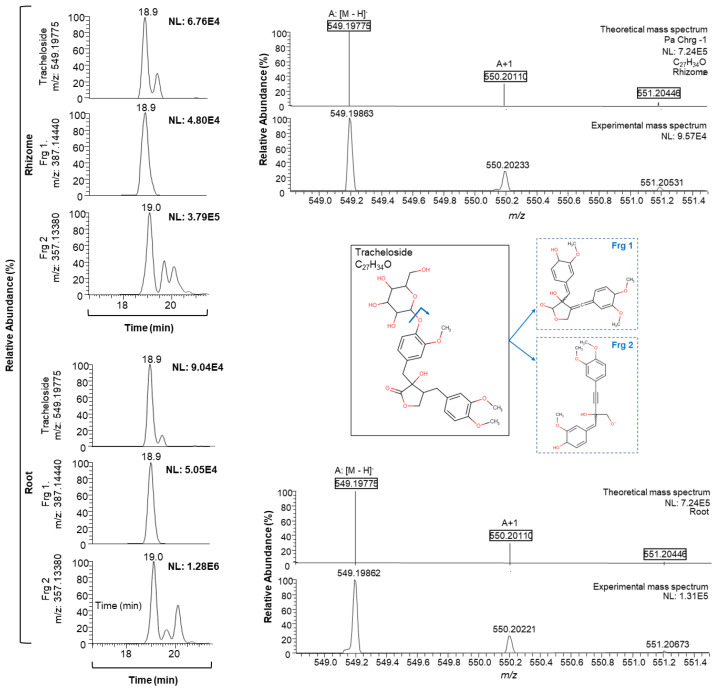
Extracted ion chromatogram of the first time detected and non-target bioactive compound tracheloside (*m/z*: 549.19775), in *P. oceanica* rhizome and root (sampling point CG4) and the corresponding mass spectrum. NL: intensity, Frg.: confirmation fragments.

Interestingly, among the first-time detected compounds, biochanin A, glycitein, gambiriin A1, and tracheloside were only observed in the underground tissues (Figure 3). To our knowledge it is also the first time that these compounds have been reported in any marine seagrass, it being more common to find them in other samples such as legumes and algae for biochanin A and glycitein, respectively [37,38].

Considering all *P. oceanica* samples and tissues, a total of 42 phenolic compounds were detected (Table S5). Among them, flavonoids and phenolic acids represented the main groups, as shown in Table 4. These two families were further investigated, revealing the cinnamic acid subclass responsible for the uneven distribution among tissues. Information on the average number of compounds per tissue is shown in Table S6. The rest of the phenolic subclasses show a relatively similar number of phenolic compounds in leaf, rhizome, and root. This difference in *P. oceanica* leaves could be ascribed to a greater exposition to UV radiation, current motion, shifts in water temperature, epiphytes load, or turbidity, among others [5,39]. For instance, marine water is relatively more unstable than sediment in terms of environmental conditions.

## 3. Material and Methods

### 3.1. Sampling

**Figure 4:**
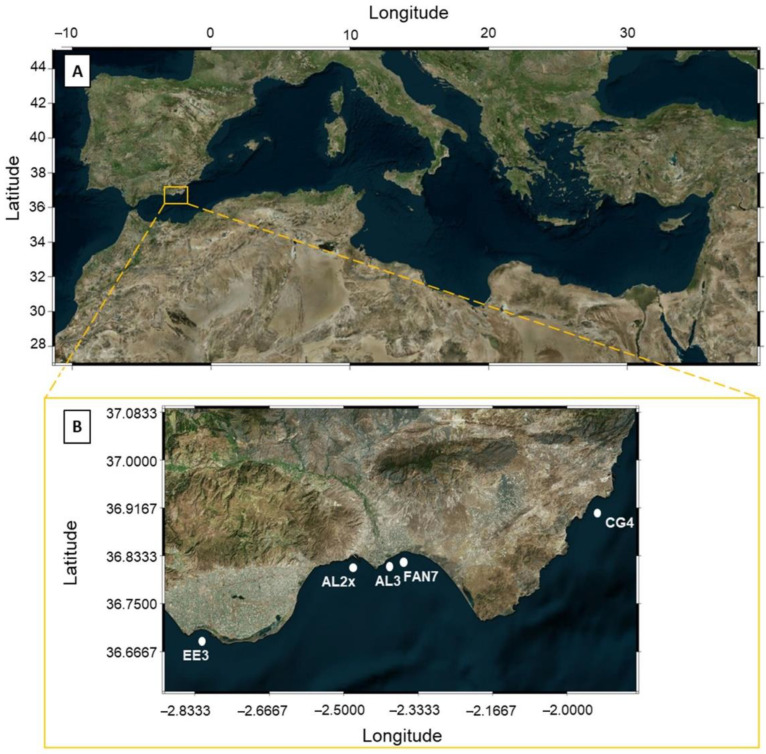
Study Area: (**A**) Map of the Mediterranean Sea Basin; the yellow square highlights Almeria coast, our study area. (**B**) Map of the coast of Almeria indicating the locations of the sampling stations. From west to east: EE3, AL2x, AL3, FAN7, and CG4. Coordinate System: WGS84, UTM.

In this study, five samples of *P. oceanica* (EE3, AL2x, AL3, FAN7, and CG4) were taken from different sites along the coast of Almeria (Alboran Sea, Spain), as can be observed in Figure 4. Samples were collected manually by scuba divers in July, October, November, December 2019, and January 2020. The sampling sites were distributed at different water depths, from 1.5 m at CG4 to 9 m at EE3. More relevant information concerning the characteristics of the sampling points can be observed in Table 5.

### 3.2. Sample Pre-Treatment

After collection, samples were immediately stored in portable fridges at a low temperature until arrival at the laboratory. Subsequently, sand and salt were removed by rinsing with distilled water and samples were divided into three parts: leaf, rhizome, and root. All parts were kept in petri plates and weighted. Note that young leaves and basal sheathes were not considered in this study.

Afterward, samples were stored at −20 °C (48 h) and freeze-dried at −50 °C (48 h) in a Thermo Electron Corporation Heto PowerDry LL3000 freeze-dryer (Thermo Fisher Scientific, Bremen, Germany). In the case of the leaves, this part was conducted in two separate steps of 24 h. In between steps, leaves were gently cleaned from epiphytes (dried crust) using a brush. Subsequently, all samples were homogenized by powdering in a Mixer Mill MM 200 (2 min at 25 r/s) and stored in desiccators until extraction.

### 3.3. Chemical and Reagents

LC/MS-grade water and methanol were purchased from Merck KGaA (Darmstadt, DE) and Riedel-de-Haën™ (Seelze, Germany), respectively. Formic acid was purchased from Fisher Scientific (Waltham, MA, USA). Galangin standard was obtained from Extrasynthese (Genay, France). P-coumaric acid, kaempferol-3-O-glucoside, quercetin standards and ammonium formate were purchased from Sigma-Aldrich (St Louis, MA, USA). All reagents were of analytical grade. Employed standards had a purity of >99%. Individual standard solutions of 300 mg l^−1^ were prepared in methanol. Resulting stock standards solutions were kept in amber bottles. Subsequently, a multi-compound working solution (50 mg l^−1^) was prepared by diluting each stock solution aliquot with methanol. All solutions were stored at 4 °C in an amber bottle until analysis.

### 3.4. Extraction Procedure

Specific extractions were developed regarding the investigated tissues from previous procedures with minor modifications [17,27]. Briefly, 3 mL of methanol/water 5:5 *v*/*v* solution (pH 4, acidified with formic acid) were added to 15 mL falcon tubes with 150 mg dry weight of leaf powder, whereas 3 mL of methanol/water 8:2 *v*/*v* solution was used in the case of rhizome and roots. All different mixtures were then sonicated for 4 min at room temperature (~25 °C) and centrifuged (5000 rpm, 10 min). One single extraction cycle was carried out since preliminary studies proved it to be adequate to monitor the phenolic compounds [27]. After ca. 15 min extraction the resulting supernatant was filtered (45 µm, Fisher Scientific, Madrid, Spain) into 2 mL LC vials and analyzed.

### 3.5. Chromatographic Conditions

Chromatographic analyses of *P. oceanica* tissues were conducted on a Thermo Scientific Transcend^TM^ 600 liquid chromatography (Thermo Fisher Scientific, San Jose, CA, USA). The employed column was Hypersil GOLD^TM^. The chromatographic separation was performed using a mobile phase that comprises water (1% formic acid and 4 mM ammonium formate) as eluent A and methanol as eluent B.

Elution from the UHPLC column gradient was carried out as follows: from 0 to 8 min, 5–30% B; from 8 to 13 min, 30–50% B; from 13 to 18 min, 50% B; from 18 to 23 min, 50–60% B; from 23 to 28 min, 60–70% B; from 28 to 33 min, 70–80% B; from 33 to 47 min, 80–100%; from 47 to 49 min, 100%; from 49 to 53.5 min, 100–10% and from 53.5 to 58 min, 10%.

The column temperature during analysis was maintained at room temperature (25 °C), the flow rate was settled at 0.2 mL min^−1^ and the injection volume at 10 µL along the process.

### 3.6. Orbitrap-MS Analysis

In this study, a single Orbitrap mass spectrometer (Exactive™, Thermo Fisher Scientific, Bremen, Germany) was used for MS analyses. The mass spectra were acquired employing four alternating acquisition functions: full MS, without fragmentation (higher collisional dissociation, HCD, collision cell was switched off), mass resolving power 25,000 FWHM (full width at half maximum); scan time 0.25 s, ESI+, and ESI−; all-ion fragmentation (AIF), with fragmentation (HCD on, collision energy 30 eV), mass resolving power 10,000 FWHM, ESI+, and ESI−. Mass range in full scan mode was set at *m/z* 100–1000, whereas, for MS/MS monitoring, it was set at *m/z* 70–700.

Data acquisition and processing were carried out using Trace Finder Version^TM^ 4.0 and Xcalibur^TM^ Version 2.2.1 (Thermo Fisher Scientific, Les Ulis, France) in Qual browser mode. Software searching criteria was set on 5 ppm mass tolerance error.

### 3.7. Phenolic Identification

As mentioned above, the identification of compounds was performed in three steps. Firstly, a target screening was carried out, submitting an in-house database containing 93 phenolic compounds to the software to elucidate the presence or absence of listed compounds. Further important information for the compound identification, such as molecular formula, retention time (*R_T_)*, exact theoretical mass of the molecular ion and characteristic fragments, and ionization mode, were also included in the in-house database.

Secondly, a suspected screening was conducted. In this case, previously detected phenolic compounds in *P. oceanica,* as well as in other Mediterranean seagrasses, were considered. This list was developed based on available data (literature and open databases such as PubChem, ChemSpider, and Human Metabolomics), adding to the study 30 suspected compounds detected in *P. oceanica* and eight only detected in the other Mediterranean seagrasses (Table 3 and Table S4). The exact mass of these suspected compounds was calculated using their chemical formula and the corresponding ionization mode (ESI−/ESI+). Confirmation fragments were also retrieved from the available literature and open databases. Note that several of the found suspected compounds were already present in our in-house database and were therefore treated as target compounds.

In third place, a non-target analysis was performed using Compound Discoverer Software (Thermo Fisher Scientific, Les Ulis, France). For that purpose, the raw data obtained from the sample and blank injections were submitted to the software. The software carried out a structural analysis, performing a metabolite profiling base on several selected databases (CheBI, KEGG, MolBank, Nature Chemistry, Sigma Aldrich, and Phenol Explorers) and the possible adducts ([M+CH_2_O_2_-H]^−1^, [M+H]^+1^, and [M-H]^−1^). Subsequently, resulting potential precursor ions and characteristic fragments were confirmed using XcaliburTM Version 2.2.1 (Thermo Fisher Scientific, Les Ulis, France) in Qual browser mode.

## 4. Conclusions

The analysis of the three *P. oceanica* tissues using, for the first time, UHPLC-Orbitrap MS in target analysis mode in combination with retrospective analyses (suspected and non-target modes) contributed to the detection of 42 phenolic compounds. Out of these, 24 have not been previously reported in this marine angiosperm. The vast majority of the detected compounds belong to the flavonoid family, highlighting the presence of flavonols and flavanones, although some phenolic acids such as cinnamic and benzoic acids were also found.

Additionally, the distribution, in terms of number of total detected phenolic compounds, was relatively higher in leaves followed by rhizome and in last place roots. It seems that the cinnamic acids subclass is responsible for such a difference (caffeic acid, caftaric acid, chicoric acid, p-coumaric acid, fertaric acid, ferulic acid, and zosteric acid). However, only through the detailed research of the three tissues the complete *P. oceanica* phenolic fingerprint was unveiled, since several compounds were merely observed in underground tissues (e.g., bioachin A, glycitein, gambrin A1, and tacharoside).

This study brings new reference information in the field of bioactive compounds in marine seagrasses, namely a comprehensive array of phenolic compounds in *P. oceanica* tissues. In addition, the number of flavonoids, compounds widely employed as biomarkers, present in *P. oceanica* has been increased. Perhaps these first-time observed flavonoids could bring lacking information in the field of seagrasses chemical reactions.

The 42 detected compounds that make up the phenolic fingerprint of *P. oceanica* should be considered in future research to observe their behavior under different conditions and further evaluate their potential as specific-biochemical markers.

## Figures and Tables

**Table 1:** Retention time (*R_T_*) and *m/z* ions for the identification and confirmation of the detected target compounds in *P. oceanica* roots (●), rhizomes (■), and/or leaf (◊). Mass error corresponds to the smallest average mass error found among the three tissues.

*R_T_* (min)	Compound Name	Elemental Composition	Polarity	Theoretical Mass (*m/z*)	Mass Error (ppm)	Fragment 1 (*m/z*)	Fragment 2 (*m/z*)	Fragment 3 (*m/z*)	Tissue
13.3	Catechin (+) [31]	C_15_H_14_O_6_	ESI+	291.08631	0.080	139.03895	123.04502		● ■ ◊
14.9	Caffeic acid [17,19]	C_9_H_8_O_4_	ESI−	179.03498	−0.127	135.04429	134.03628	89.03847	● ■ ◊
16.1	Epicatechin (−) [31]	C_15_H_14_O_6_	ESI+	291.08631	0.122	139.03895	123.04502		● ■ ◊
16.3	Genistein *	C_15_H_10_O_5_	ESI+	271.06010	0.189	153.01779	215.06962	243.06434	● ■ ◊
16.3	Baicalein *	C_15_H_10_O_5_	ESI+	271.06010	0.056	253.04950	243.06520		● ■ ◊
16.9	Eriodictyol *	C_15_H_12_O_6_	ESI−	287.05611	1.633	151.00235	107.01253		● ■ ◊
17.6	p-Coumaric acid [8,15]	C_9_H_8_O_3_	ESI−	163.04007	−0.362	119.04881	93.03316	163.03950	● ■ ◊
18.1	Ferulic Acid [16,31]	C_10_H_10_O_4_	ESI−	193.05063	−1.482	134.03643	149.06100	178.02640	● ■ ◊
19.6	Quercetin-3-O-glucoside [14]	C_21_H_20_O_12_	ESI−	463.08710	0.821	300.02700	302.03696	301.03455	● ■ ◊
20.7	Kaempferol-3-O-glucoside * + Luteolin-4’-O-glucoside *	C_21_H_20_O_11_	ESI−	447.09328	1.939	284.03200	255.02924	285.03995	● ■ ◊
20.8	Isorhamnetin-3-O-glucoside [14]	C_22_H_22_O_12_	ESI+	479.11840	0.646	317.06550			● ■ ◊
22.4	Quercetin [7]	C_15_H_10_O_7_	ESI+	303.04993	0.001	201.05453	153.01834	165.01837	● ■
23.2	Naringenin *	C_15_H_12_O_5_	ESI−	271.06012	0.271	119.04879	151.00226	107.01350	● ■ ◊
23.4	Luteolin *	C_15_H_10_O_6_	ESI−	285.04046	0.269	133.02834	151.00260	175.03898	● ■ ◊
26.3	Isorhamnetin [7]	C_16_H_12_O_7_	ESI−	315.05103	2.013	300.02685	151.00245		● ■ ◊
26.5	Apigenin *	C_15_H_10_O_5_	ESI+	271.06010	−0.037	153.01779	119.04943		◊
27.4	Naringenin Chalcone *	C_15_H_12_O_5_	ESI−	271.06012	0.281	119.04879	151.00226	107.01350	● ■ ◊
30.6	Pinocembrin *	C_15_H_12_O_4_	ESI−	255.06628	0.241	151.00241	213.05467		● ■ ◊
30.7	Biochanin A *	C_16_H_12_O_5_	ESI−	283.06120	1.142	268.03634			● ■
30.7	Glycitein *	C_16_H_12_O_5_	ESI+	285.07575	−0.667	270.05097	242.05613		● ■
33.3	Galangin *	C_15_H_10_O_5_	ESI−	269.04555	0.068	213.05450			● ◊
34.4	Sakuranetin * + Isosakuranetin *	C_16_H_14_O_5_	ESI−	285.07685	1.844	119.04883	221.15330	165.01802	● ■ ◊

* Compounds not detected before in *P. oceanica*.

**Table 2:** Retention time (*R_T_*) and *m/z* ions of the detected suspected compounds in the Orbitrap system for *P. oceanica* and other Mediterranean seagrasses (@): *Cymodocea nodosa* and *Zostera marina* and *Zostera noltii*. Compounds detected in *P. oceanica* roots (●) rhizomes (■), and/or leaves (◊).

*R_T_* (min)	Compound Name	Elemental Composition	Polarity	Theoretical Mass (*m/z*)	Mass Error (ppm)	Fragment 1 (*m/z*)	Fragment 2 (*m/z*)	Fragment 3 (*m/z*)	Tissue	Reference
7.6	Protocatechualdehyde	C_7_H_6_O_3_	ESI−	137.02442	−4.850	136.01660	108.02050	109.03050	● ■ ◊	[16]
13.1	Zosteric acid@	C_9_H_8_O_6_S	ESI−	242.99688	0.371	163.04010	145.02950	117.03460	● ■ ◊	[19,35]
16.1	p-Anisic acid	C_8_H_8_O_3_	ESI−	151.04007	−1.210	133.02861	123.04398		● ■ ◊	[16]
16.3	Caftaric Acid	C_13_H_12_O_9_	ESI−	311.04086	1.437	130.99800	161.02390	267.05050	● ■ ◊	[17]
16.3	Chicoric acid	C_22_H_18_O_12_	ESI−	473.07255	1.672	311.04071	293.02844	149.00810	● ■ ◊	[14,19]
17.7	Fertaric acid	C_14_H_14_O_9_	ESI−	325.05651	1.658	193.05010	130.99800	87.00820	■ ◊	[17]
18.3	Cinnamic Acid	C_9_H_8_O_2_	ESI−	147.04515	−0.713	119.04916	117.03351	101.03851	● ■	[15]
19.9	Quercetin-3-O-malonylglucoside	C_24_H_22_O_15_	ESI−	549.08859	1.007	505.10006	300.02737	301.03183	● ■ ◊	[14]
21.3	Isorhamnetin-3-O-malonylglucoside	C_25_H_24_O_15_	ESI−	563.10424	2.148	459.09270	315.05050	299.01920	● ■ ◊	[14]

**Table 3:** Average retention times (*R_T_*) and *m/z* ions of the non-target compounds in the Orbitrap system for *P. oceanica*. Compounds detected in *P. oceanica* roots (●) rhizomes (■), and/or leaves (◊).

*R_T_* (min)	Compound Name	Elemental Composition	Polarity	Theoretical Mass (*m/z*)	Mass Error (ppm)	Fragment 1 (*m/z*)	Fragment 2 (*m/z*)	Fragment 3 (*m/z*)	Fragment 4 (*m/z*)	Tissue
13.3	Gambiriin A1	C_30_H_28_O_12_	ESI−	579.15080	2.881	125.02390	289.07120	151.03950	139.03950	● ■
18.1	Mascaroside	C_26_ H_36_ O_11_	ESI−	523.21849	1.447	361.16510	331.15450			● ■ ◊
18.3	Astilbin	C_21_H_22_O_11_	ESI−	449.10893	1.575	151.00322	150.03022	303.05050	285.03990	● ■ ◊
18.9	Tracheloside	C_27_ H_34_ O_12_	ESI−	549.19775	1.640	387.14440	357.13380			● ■
21.0	Quercetin 3-O-sulfate	C_15_H_10_O_10_S	ESI−	380.99219	0.372	301.03480	80.96460			● ■ ◊
33.7	Glabridin	C_20_H_20_O_4_	ESI−	323.12888	1.317	187.07590	267.06570			◊
34.5	Piceatannol	C_14_H_12_O_4_	ESI+	245.08084	0.002	135.04460	215.07080	227.07080		● ■ ◊
39.1	Sophoraflavanone B	C_20_H_20_O_5_	ESI−	339.12380	1.093	219.06418	119.04871			● ■ ◊
40.7	Tetrahydrocurcumin	C_21_H_24_O_6_	ESI−	372.15790	0.637	177.05520	193.08650	219.06570		● ■ ◊
41.8	Demethoxycurcumin	C_20_ H_18_ O_5_	ESI−	337.10815	1.836	119.04970	161.06030	175.03950	217.05010	● ■ ◊
45.3	Xanthohumol	C_21_H_22_O_5_	ESI−	353.13945	1.155	207.10210	119.04900	145.02900		● ■ ◊

**Table 4:** Phenolic families detected in *P. oceanica* tissues and the maximum number observed in the plant.

		Flavonoids	Phenolic Acids	Other Polyphenols	Total Number
EE3	Leaf	20	9	3	32
	Rhizome	22	5	3	30
	Root	22	5	3	30
FAN7	Leaf	19	9	3	31
	Rhizome	24	5	3	32
	Root	20	4	3	27
AL2x	Leaf	21	9	3	33
	Rhizome	21	5	3	29
	Root	21	5	3	29
AL3	Leaf	22	9	2	33
	Rhizome	21	5	2	28
	Root	19	5	2	26
CG4	Leaf	23	8	3	34
	Rhizome	23	5	3	31
	Root	24	5	3	32
	Maximum	24	9	3	34

**Table 5:** Characteristics of the considered sampling points in Almeria (Spain).

Site	Water Depth (m)	Location (WGS84_UTM)	Distance from Coast (m)	Area Description	Sampling Date
EE3	10.3	36.682721, −2.781700	670	Limit between a harbor and a nature spot. Influenced by watershed with intensive agriculture (greenhouses)	31 Oct 2019
AL2x	7.8	36.824655, −2.452103	80	Touristic city, harbor	18 Dec 2019
AL3	7.8	36.828547, −2.385920	540	Sewage, airport, and watercourse (seasonal)	11 Nov 2019
FAN7	-	36.835713, −2.352617	-	Submarine natural gas pipeline (MEDGAZ) and watercourse (seasonal)	2 Jul 2019
CG4	1.5	36.862794, −2.003661	5	Marine Protected Area	5 Jan 2020

## Data Availability

Data is contained within the article.

## Supplementary Materials

The following are available online at https://www.mdpi.com/article/10.3390/plants10050864/s1, Table S1: Compounds and their corresponding average intensities detected during the extractant composition evaluation (methanol/water, 5:5 *v*/*v* and 8:2 *v*/*v*) using *P. oceanica* tissues from FAN7 sampling point; Table S2: Traditional and IUPAC names of the first-time detected compounds in *P. oceanica* samples; Table S3: Biological properties of some bioactive compounds found in *P. oceanica* tissues; Table S4: List of suspected compounds retrieved from available literature for *P. oceanica* and other seagrasses (such as *Cymodocea nodosa*, *Zostera marina*, and *Zostera noltii*) that were not detected in this study. Theoretical mass in ESI- and ESI+ mode is provided for those compounds that were not present in our in-house database; Table S5: List of the phenolic compounds detected in *P. oceanica*, their precursor ions (Prc. Ion) and confirmation fragments (Frg.) in each tissue and sampling point; Table S6: Total phenolic compounds detected in *P. oceanica* tissues (target, suspected, and non-target) and the maximum number observed in the plant (grey). Subclasses from left to right: cinnamic acids (CA), benzoic acids (BA), flavones (FL), flavonols (FLL), isoflavones (i-FL), flavonons/flavanones (FLN), chalcones (CHL), catechins (CT), prenylated isoflavonoids (pr-IsF), dihydroflavonols (d-FLL), curcuminoids (CU), and stilbenes (ST); Figure S1: Extracted ion chromatogram for naringenin and naringenin chalcone (271.06012 *m*/*z*) in full MS of FAN7 leaves using Acquity C18 column and Hypersil GOLD column. NL: intensity; Figure S2: Extracted ion chromatogram for ferulic acid (full MS, *m*/*z*: 193.05063) and its confirmation fragment (MS/MS, *m*/*z*: 134.03643) in: (A) ammonium acetate and (B) formic acid as mobile phase. *R_T_*: 17.9 min. NL: intensity; and Figure S3: Workflow indicating the conducted steps for the complete phenolic compound screening in the marine seagrass *P. oceanica*. Roots (●), rhizomes (■), and leaf (◊).
