# Supplementary material for: New Phenolic Compounds in Posidonia oceanica Seagrass: A Comprehensive Array Using High Resolution Mass Spectrometry
Source: Plants (Basel). 2021 Apr 25;10(5):864. doi: 10.3390/plants10050864 (PMC8145229; doi:10.3390/plants10050864)
Supplement: Supplementary file 1 [file plants-10-00864-s001.zip › plants-1193662-supplementary.pdf]

Supplementary material

New phenolic compounds in *Posidonia oceanica* seagrass: a comprehensive array using high resolution mass spectrometry

Marina Astudillo-Pascual[†], Irene Domínguez[*, ‡], Pedro A. Aguilera[†] and Antonia Garrido Frenich[‡]

[†]*Department of Biology and Geology, International Campus of Excellence in Marine Science (CEIMAR), University of Almería, E-04120 Almería, Spain*

[‡]*Department of Chemistry and Physics, Research Centre for Mediterranean Intensive Agrosystems and Agri-Food Biotechnology (CIAIMBITAL), Agrifood Campus of International Excellence, ceiA3. University of Almería, E-04120 Almería, Spain*

Table S1. Compounds and their corresponding average intensities detected during the extractant composition evaluation (methanol/water, 5:5 *v/v* and 8:2 *v/v*) using *P. oceanica* tissues from FAN7 sampling point.

	**Leaf**		**Rhizome**		**Root**	
**Target compounds**	**5:5**	**8:2**	**5:5**	**8:2**	**5:5**	**8:2**
Baicalein	3.7E4	-	7.39E4	7.78E4	-	-
Biochanin A	-	-	1.5E4	1.2E5	7.0E3	5.0E4
Caffeic acid	2.3E6	6.2E5	2.5E4	5.2E4	3.2E4	6.9E4
Catechin (+)	1.9E5	9.8E4	2.0E6	2.9E6	9.0E4	1.9E5
p-Coumaric acid	1.1E7	6.4E6	3.7E5	1.1E6	1.2E5	2.9E5
Epicatechin (-)	2.2E5	6.0E4	1.2E6	1.9E6	5.8E4	1.5E5
Eriodictyol	3.1E3	2.6E3	4.3E4	1.0E5	3.1E3	1.4E4
Ferulic acid	4.5E6	1.5E5	2.0E4	5.1E4	2.8E4	6.9E4
Galangin	3.2E4	5.3E4	-	-	2.4E3	1.7E4
Genistein	3.7E4	-	7.4E4	7.8E4	-	-
Glycitein	-	-	-	1.2E5	-	3.6E4
Isorhamnetin	1.0E4	5.6E5	2.1E3	3.6E4	7.1E3	1.9E5
Isorhamnetin-3-O-glucoside	3.3E6	2.2E6	2.9E5	7.7E5	2.0E5	5.9E5
Sakuranetin + Isosakuranetin	3.7E6	4.0E6	4.9E3	1.9E4	8.1E3	3.7E4
Kaempferol-3-O-glucoside + Luteolin-4'-O-glucoside	3.8E5	2.9E5	4.1E4	1.2E5	1.3E4	4.0E4
Luteolin	1.3E4	1.8E4	-	5.7E3	1.0E3	1.2E4
Naringenin	3.3E5	2.3E5	1.5E4	4.8E4	3.6E4	2.0E5
Naringenin Chalcone	2.3E5	1.9E5	2.4E3	6.1E3	1.5E4	8.4E4
Pinocembrin	4.4E5	3.7E5	1.1E4	3.4E4	4.6E4	2.3E5
Quercetin	-	-	-	3.4E4	-	-

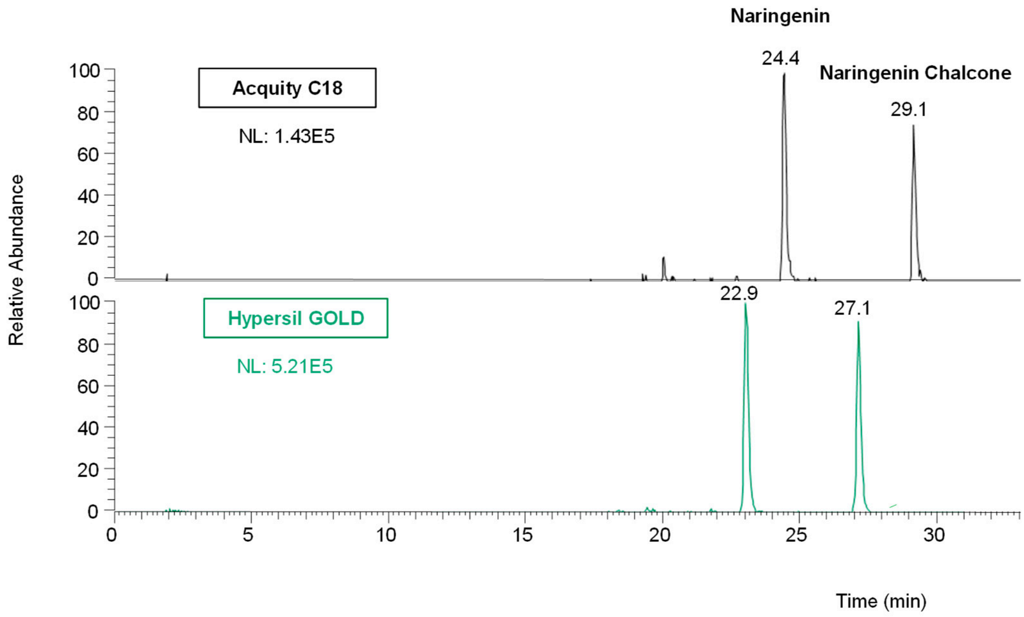

Figure S1. Extracted ion chromatogram for naringenin and naringenin chalcone (271.06012 *m/z*) in full MS of FAN7 leaves using Acquity C18 column and Hypersil GOLD column. NL: intensity.

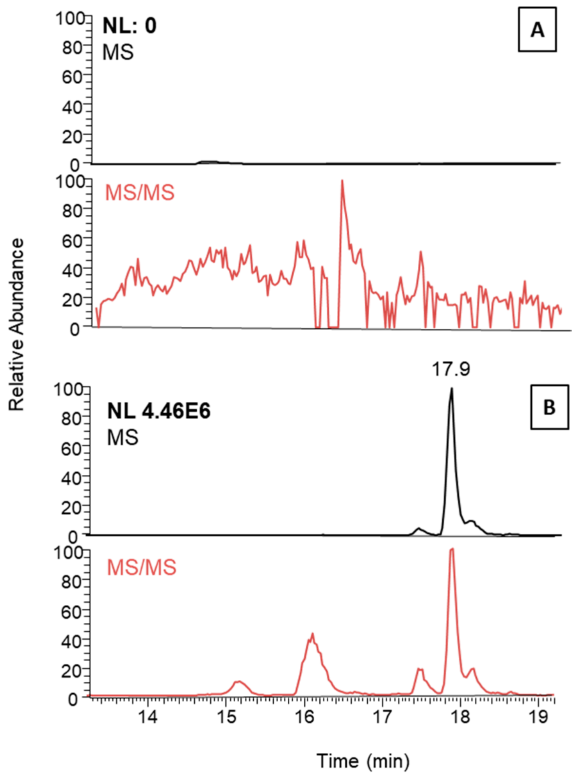

Figure S2. Extracted ion chromatogram for ferulic acid (full MS, *m/z*: 193.05063) and its confirmation fragment (MS/MS, *m/z*: 134.03643) in: (A) ammonium acetate and (B) formic acid as mobile phase. R_T: 17.9 min. NL: intensity.

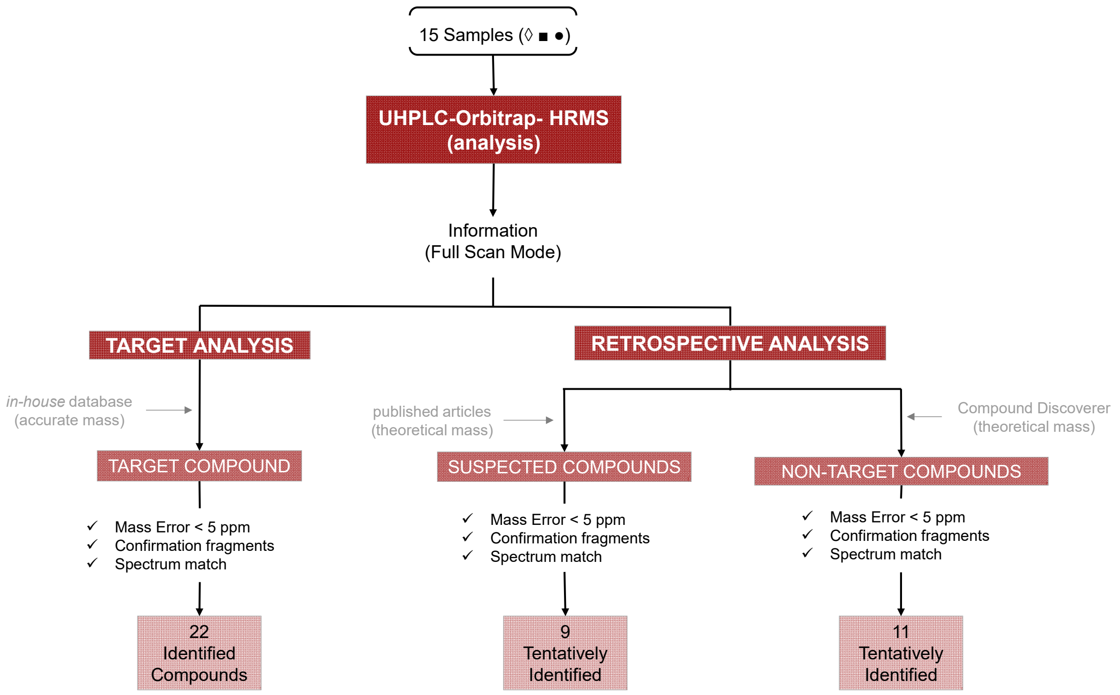

Figure S3. Workflow indicating the conducted steps for the complete phenolic compound screening in the marine seagrass *P. oceanica*. Roots (●), rhizomes (■), and leaf (◊).

Table S2. Traditional and IUPAC names of the first-time detected compounds in *P. oceanica* samples.

Traditional	IUPAC
Astilbin	(2R,3R)-2-(3,4-dihydroxyphenyl)-5,7-dihydroxy-3-[(2S,3R,4R,5R,6S)-3,4,5-trihydroxy-6-methyloxan-2-yl]oxy-2,3-dihydrochromen-4-one
Apigenin	5,7-dihydroxy-2-(4-hydroxyphenyl)-4H-chromen-4-one
Baicalein	5,6,7-trihydroxy-2-phenyl-4H-chromen-4-one
Biochanin A	5,7-dihydroxy-3-(4-methoxyphenyl)-4H-chromen-4-one
Demethoxycurcumin	(1E,6E)-1-(4-hydroxy-3-methoxyphenyl)-7-(4-hydroxyphenyl)hepta-1,6-diene-3,5-dione
Eriodictyol	(2S)-2-(3,4-dihydroxyphenyl)-5,7-dihydroxy-3,4-dihydro-2H-1-benzopyran-4-one
Galangin	3,5,7-trihydroxy-2-phenyl-4H-chromen-4-one
Gambiriin A1	(2R,3S)-2-(3,4-dihydroxyphenyl)-8-[(1R,2S)-1-(3,4-dihydroxyphenyl)-2-hydroxy-3-(2,4,6-trihydroxyphenyl)propyl]-3,4-dihydro-2H-chromene-3,5,7-triol
Genistein	5,7-dihydroxy-3-(4-hydroxyphenyl)-4H-chromen-4-one
Glabridin	4-[(3R)-8,8-dimethyl-3,4-dihydro-2H-pyrano[2,3-f]chromen-3-yl]benzene-1,3-diol
Glycitein	7-hydroxy-3-(4-hydroxyphenyl)-6-methoxy-4H-chromen-4-one
Isosakuranetin	(2S)-5,7-dihydroxy-2-(4-methoxyphenyl)-2,3-dihydrochromen-4-one
Kaempferol-3-O-glucoside	5,7-dihydroxy-2-(4-hydroxyphenyl)-3-{[(2S,3R,4S,5S,6R)-3,4,5-trihydroxy-6-(hydroxymethyl)oxan-2-yl]oxy}-4H-chromen-4-one
Luteolin	2-(3,4-dihydroxyphenyl)-5,7-dihydroxy-4H-chromen-4-one
Luteolin-4'-O-glucoside	5,7-dihydroxy-2-(3-hydroxy-4-{[3,4,5-trihydroxy-6-(hydroxymethyl)oxan-2-yl]oxy}phenyl)-4H-chromen-4-one
Mascaroside	(1R,4S,12R,13S,14S,16S,17R,18R)-14,17,18-trihydroxy-12-methyl-17-[[(2R,3R,4S,5S,6R)-3,4,5-trihydroxy-6-(hydroxymethyl)oxan-2-yl]oxymethyl]-8-oxapentacyclo[14.2.1.01,13.04,12.05,9]nonadeca-5(9),6-dien-10-one
Naringenin	(2S)-5,7-dihydroxy-2-(4-hydroxyphenyl)-3,4-dihydro-2H-1-benzopyran-4-one
Naringenin Chalcone	3-(4-hydroxyphenyl)-1-(2,4,6-trihydroxyphenyl)prop-2-en-1-one
Pinocembrin	(2S)-5,7-dihydroxy-2-phenyl-3,4-dihydro-2H-1-benzopyran-4-one
Quercetin 3-O-sulfate	[2-(3,4-dihydroxyphenyl)-5,7-dihydroxy-4-oxochromen-3-yl] hydrogen sulfate
Sophoraflavanone B	5,7-dihydroxy-2-(4-hydroxyphenyl)-8-(3-methylbut-2-enyl)-2,3-dihydrochromen-4-one
Sakuranetin	(2S)-5-hydroxy-2-(4-hydroxyphenyl)-7-methoxy-3,4-dihydro-2H-1-benzopyran-4-one
Tetrahydrocurcumin	1,7-bis(4-hydroxy-3-methoxyphenyl)heptane-3,5-dione

Tracheloside	(3S,4S)-4-[(3,4-dimethoxyphenyl)methyl]-3-hydroxy-3-[[3-methoxy-4-[(2S,3R,4S,5S,6R)-3,4,5-trihydroxy-6-(hydroxymethyl)oxan-2-yl]oxyphenyl]methyl]oxolan-2-one
trans-Piceatannol	4-[2-(3,5-dihydroxyphenyl)ethenyl]benzene-1,2-diol
Xanthohumol	(E)-1-[2,4-dihydroxy-6-methoxy-3-(3-methylbut-2-enyl)phenyl]-3-(4-hydroxyphenyl)prop-2-en-1-one

Table S3. Biological properties of some bioactive compounds found in *P. oceanica* tissues.

Flavones and flavonols	anti-inflammatory, antigenotoxic, and antihyperglycemic
Isoflavones	anti-cancerous, antioxidant, anti-inflammatory, vasodilator and relieve symptoms of menopause
Flavonons and flavanones	anti-inflammatory
Chalcones	antibacterial and antioxidant
Cinnamic acids	antioxidant, antigenotoxic, and antiproliferative
Benzoic acids	protective effect against liver damage

Table S4. List of suspected compounds retrieved from available literature for *P. oceanica* and other seagrasses (such as *Cymodocea nodosa*, *Zostera marina* and *Zostera noltii)* that were not detected in this study. Theoretical mass in ESI- and ESI+ mode is provided for those compounds that were not present in our *in-house* database.

**Compound Name**	**Elemental Composition**	**Theoretical mass (*m/z*) with ESI-**	**Theoretical mass (*m/z*) with ESI+**	***P. oceanica***	**others**
Acetovallinone	$C_9H_{10}O_3$	165.05572	167.07027	x	x
Acetosyringone	$C_{10}H_{12}O_4$	195.06628	197.08084	x	
(methyl 12-) Acetoxyricinoleate	$C_{21}H_{38}O_5$	369.26465	371.27920	x	
Apigenin 7-O-glucoside	$C_{21}H_{20}O_{10}$		433.11292		x
Apigenin 7-sulfate	$C_{15}H_{10}O_8S$	349.00236	351.01691		x
Acacetin-7-sulfate	$C_{16}H_{11}O_9S$	378.00510	380.01965		x
Campestanol	$C_{28}H_{50}O$	401.37889	412.46397	x	
Chlorogenic acid	$C_{16}H_{18}O_9$		355.10236	x	
Coutaric acid	$C_{13}H_{12}O_8$	295.04594	297.06049	x	
Diosmetin 7-sulfate	$C_{16}H_{11}O_9S$	379.01293	381.02748		x
Gallic acid	$C_7H_6O_5$	169.01315		x	x
Gentisic acid	$C_7H_6O_4$	153.01933	155.03389	x	
4-Hydroxybenzaldehyde	$C_7H_6O_2$	121.0295	123.04406	x	
4-Hydroxybenzoic acid	$C_7H_6O_3$	137.02332		x	
Kaempferol	$C_{15}H_{10}O_6$		287.05501	x	
Luteolin 7-O-glucoside	$C_{21}H_{20}O_{11}$	447.09219			x
Luteolin 7-sulfate	$C_{15}H_{10}O_9S$	364.99728	367.01183		x
Myricetin	$C_{15}H_{10}O_8$	317.02919		x	
Phloroglucinol	$C_6H_6O_3$	125.02442	127.03897	x	
Piccol	$C_8H_8O_2$	135.04406	137.05971	x	

Proanthocyanidin	$C_{30}H_{26}O_{13}$	593.13006	595.14462		x
Procyanidin B2	$C_{30}H_{26}O_{12}$	577.13515	579.14970	x	
Procyanidin C2	$C_{45}H_{38}O_{18}$	865.19854	867.21309	x	
Protocatechuic acid	$C_7H_6O_4$	153.01933	155.03389	x	
Pyrocatechol	$C_6H_6O_2$	109.02950	111.04406	x	
Pyrogallol	$C_6H_6O_3$	125.02442	127.03897	x	
Rosmarinic acid	$C_{18}H_{16}O_8$	359.07724	361.09179		x
Sinapic acid	$C_{11}H_{12}O_5$	223.06120		x	
Syringaldehyde	$C_9H_{10}O_4$	181.05063	183.06519	x	x
Syringic acid	$C_9H_{10}O_5$	197.04555		x	
diferuloyl Tartaric acid	$C_{24}H_{22}O_{12}$	501.10385	503.11840	x	
dicoumaroyl Tartaric acid	$C_{22}H_{18}O_{10}$	441.08272	443.09727	x	
Vanillic acid	$C_8H_8O_4$	167.03498		x	
Vanillin	$C_8H_8O_3$	151.03897	153.05462	x	x

Table S5. List of the phenolic compounds detected in *P. oceanica*, their precursor ions (Prc. Ion) and confirmation fragments (Frg.) in each tissue and sampling point.

				Average Mass Error (ppm)			Accurate *m/z*														
							Point AL2x			Point EE3			Point AL3			Point CG4			Point FAN7		
**Compound**	**R_T** (average± SD)	**Ions**	**Theoretical mass (*m/z*)**	**Leaf (◊)**	**Rhiz (■)**	**Root (●)**	◊	■	●	◊	■	●	◊	■	●	◊	■	●	◊	■	●
Protocatechualdehyde $C_7H_6O_3$ ESI-	7.6 ± 0.12	Prc. Ion	137.02442	-4.980	-4.956	-4.850	x	x	x	x	x	x	x	x	x	x	x	x	x	x	x
		Frg 1.	136.01660				x	x		x	x		x	x		x	x	x	x	x	x
		Frg 2.	108.02050				x	x		x			x	x		x			x	x	x
		Frg 3.	109.03050																		
Zosteric acid $C_9H_8O_6S$ ESI-	13.1 ± 0.08	Prc. Ion	242.99688	-0.049	0.371	0.642	x	x	x	x	x	x	x	x	x	x	x	x	x	x	x
		Frg 1.	163.04010				x	x	x	x	x	x	x	x	x	x	x	x		x	
		Frg 2.	145.02950					x			x	x		x			x	x			
		Frg 3.	117.03460								x			x			x	x			
Gambiriin A1 $C_{30}H_{28}O_{12}$ ESI-	13.3 ± 0.04	Prc. Ion	579.15080	-	2.881	4.534		x	x		x			x	x		x	x		x	
		Frg 1.	125.02390					x			x			x	x		x			x	
		Frg 2.	289.07120					x			x			x			x			x	
		Frg 3.	151.03950					x	x		x			x	x		x			x	
Catechin (+) $C_{15}H_{14}O_6$ ESI+	13.3 ± 0.07	Prc. Ion	291.08631	0.182	0.080	0.424	x	x	x	x	x	x	x	x	x	x	x	x	x	x	x
		Frg 1.	139.03895				x	x	x	x	x	x	x	x	x	x	x	x	x	x	x
		Frg 2.	123.04502				x	x	x	x	x	x	x	x	x	x	x	x	x	x	x
Caffeic acid $C_9H_8O_4$ ESI-	14.9 ± 0.04	Prc. Ion	179.03498	-0.127	1.341	-0.558	x	x	x	x	x	x	x	x	x		x	x	x	x	x
		Frg 1.	135.04429				x		x	x	x	x	x		x						x
		Frg 2.	134.03628				x	x	x	x	x	x	x	x	x		x	x	x	x	
		Frg 3.	89.03847				x		x	x		x	x		x						

p-Anisic acid $C_8H_8O_3$ ESI-	16.1 ± 0.05	Prc. Ion	151.04007	-1.328	-1.262	-1.210	x	x	x	x	x	x	x	x	x	x	x	x	x	x	x
		Frg 1.	133.02861					x		x		x	x	x		x	x			x	x
		Frg 2.	123.04398				x	x	x		x	x	x		x		x	x		x	x
Epicatechin (-) $C_{15}H_{14}O_6$ ESI+	16.1 ± 0.02	Prc. Ion	291.08631	2.003	0.122	1.221	x	x	x	x	x	x	x	x	x	x	x	x	x	x	x
		Frg 1.	139.03895				x		x		x	x		x	x		x	x		x	
		Frg 2.	123.04502				x		x		x	x	x	x	x		x	x	x	x	x
Caftaric Acid $C_{13}H_{12}O_9$ ESI-	16.3 ± 0.04	Prc. Ion	311.04086	1.437	2.394	1.649	x	x	x	x	x	x	x	x	x	x	x	x	x	x	x
		Frg 1.	130.99800				x		x	x			x		x	x		x	x	x	
		Frg 2.	161.02390				x		x	x		x	x		x	x		x	x		x
		Frg 3.	267.05050				x			x			x			x			x		
Baicalein $C_{15}H_{10}O_5$ ESI+	16.3 ± 0.03	Prc. Ion	271.06010	-1.162	0.056	0.554	x	x	x	x	x	x	x	x	x	x	x	x	x	x	
		Frg 1.	253.04950				x	x			x			x			x			x	
		Frg 2.	243.06520				x	x	x	x	x	x	x	x	x	x	x	x	x	x	
Chicoric acid $C_{22}H_{18}O_{12}$ ESI-	16.3 ± 0.04	Prc. Ion	473.07255	1.672	1.957	1.748	x	x	x	x	x	x	x	x	x	x	x	x	x	x	x
		Frg 1.	311.04071				x		x	x		x	x		x	x		x	x		x
		Frg 2.	293.02844				x	x	x	x		x	x	x	x	x	x	x	x	x	x
		Frg 3.	149.00812				x	x	x	x	x	x	x	x	x	x	x	x	x	x	x
Genistein $C_{15}H_{10}O_5$ ESI+	16.3 ± 0.03	Prc. Ion	271.06010	-1.157	0.189	0.983	x	x	x	x	x	x	x	x	x	x	x	x	x	x	
		Frg 1.	153.01779				x	x	x		x	x	x	x	x	x	x	x	x	x	
		Frg 2.	215.06962				x	x	x	x	x	x	x	x	x	x	x	x	x	x	
		Frg 3.	243.06434				x	x	x	x	x	x	x	x	x	x	x	x	x	x	
Eriodictyol $C_{15}H_{12}O_6$ ESI-	16.9 ± 0.01	Prc. Ion	287.05611	3.131	1.633	1.903					x	x	x	x	x	x	x	x	x	x	x
		Frg 1.	135.04382																		
		Frg 2.	151.00235																		
		Frg 3.	107.01253								x	x					x	x	x	x	
		Prc. Ion	163.04007	-0.574	-0.362	-0.543	x	x	x	x	x	x	x	x	x	x	x	x	x	x	x

p-Coumaric acid<br>$C_9H_8O_3$<br>ESI-	17.6 ± 0.05	Frg 1.	119.04881				x	x	x	x	x	x	x	x	x	x	x	x	x	x	x
		Frg 2.	93.03316				x	x	x	x		x	x	x	x	x			x	x	
		Frg 3.	163.03950							x	x	x	x	x	x	x	x	x	x	x	x
Fertaric acid<br>$C_{14}H_{14}O_9$<br>ESI-	17.7 ± 0.03	Prc. Ion	325.05651	1.658	-	3.691	x		x	x		x	x		x	x		x	x		
		Frg 1.	193.05010				x		x	x			x		x	x			x		
		Frg 2.	130.99800				x		x	x			x		x	x			x		
		Frg 3.	87.00820				x		x	x			x		x	x			x		
Ferulic acid<br>$C_{10}H_{10}O_4$<br>ESI-	18.1 ± 0.07	Prc. Ion	193.05063	-1.855	-1.482	-2.052	x	x	x	x	x	x	x	x	x	x	x	x	x	x	x
		Frg 1.	134.03643				x	x	x	x	x	x	x	x	x	x	x	x	x	x	x
		Frg 2.	149.06100				x	x	x	x	x	x	x	x	x	x	x	x	x	x	x
		Frg 3.	178.02640				x		x	x	x		x		x	x		x	x		
Mascaroside<br>$C_{26}H_{36}O_{11}$<br>ESI-	18.1 ± 0.03	Prc. Ion	523.21849	1.481	1.447	1.550	x	x	x	x	x	x	x	x	x	x	x	x	x	x	x
		Frg 1.	361.16510				x	x	x	x	x	x	x	x	x	x	x	x	x	x	x
		Frg 2.	331.15450					x	x		x	x		x	x		x			x	x
Astilbin<br>C21 H22 O11<br>ESI-	18.3 ± 0.03	Prc. Ion	449.10893	2.150	1.575	2.080	x	x	x	x	x	x	x	x	x	x	x	x		x	
		Frg 1.	151.00322				x	x	x		x	x	x	x	x	x	x	x		x	
		Frg 2.	150.03022				x		x		x	x			x		x	x			
		Frg 3.	303.05050										x								
Cinnamic acid<br>C9H8O2<br>ESI-	18.3 ± 0.01	Prc. Ion	147.04515	-	-0.713	0.730														x	x
		Frg 1.	119.04916																	x	x
		Frg 2.	117.03351																	x	x
		Frg 3.	101.03851																		x
Tracheloside<br>$C_{27}H_{34}O_{12}$<br>ESI-	18.9 ± 0.02	Prc. Ion	549.19775	-	1.640	1.712		x	x		x	x		x	x		x	x		x	x
		Frg 1.	387.14440					x	x		x	x		x	x		x	x		x	x
		Frg 2.	357.13380					x	x		x	x		x	x		x	x		x	x
		Prc. Ion	463.08765	0.821	1.044	1.054	x	x	x	x	x	x	x	x	x	x	x	x	x	x	x

Quercetin-3-O-glucoside $C_{21}H_{20}O_{12}$ ESI-	19.6 ± 0.02	Frg 1.	300.02700				x	x	x	x	x	x	x	x	x	x	x	x	x	x	x
		Frg 2.	302.03696				x	x	x	x	x	x	x	x	x	x	x	x	x	x	x
		Frg 3.	301.03455				x	x	x	x	x	x	x	x	x	x	x	x	x	x	x
Quercetin-3-O-malonylglucoside $C_{24}H_{22}O_{15}$ ESI-	19.9 ± 0.03	Frg 4.	549.08859	1.007	1.082	1.907	x	x	x	x	x	x	x	x	x	x	x	x	x	x	x
		Frg 1.	505.10006				x	x	x	x	x	x	x	x	x	x	x	x	x	x	x
		Frg 2.	300.02737				x	x	x	x	x	x	x	x	x	x	x	x	x	x	
		Frg 3.	301.03183				x	x		x	x		x	x	x	x	x	x	x	x	
Kaempferol-3-O-glucoside + 4. Luteolin-4'-O-glucoside $C_{21}H_{20}O_{11}$ ESI-	20.7 ± 0.03	Prc. Ion	447.09328	1.939	2.204	1.846	x	x	x	x	x	x	x	x	x	x	x	x	x	x	x
		Frg 1.	284.03200				x	x	x	x	x	x	x	x	x	x	x	x	x	x	x
		Frg 2.	255.02924				x	x	x	x	x	x	x	x	x	x	x	x	x	x	x
		Frg 3.	285.03995				x	x	x	x	x	x	x	x	x	x	x	x	x	x	x
Isorhamnetin-3-O-glucoside $C_{22}H_{22}O_{12}$ ESI+	20.8 ± 0.02	Prc. Ion	479.11840	0.759	0.880	0.646	x	x	x	x	x	x	x	x	x	x	x	x	x	x	x
		Frg 1.	317.06550				x	x	x	x	x	x	x	x	x	x	x	x	x	x	x
Quercetin 3-O-sulfate $C_{15}H_{10}O_{10}S$ ESI-	21.0 ± 0.03	Prc. Ion	380.99219	0.778	0.854	0.372		x	x	x	x	x	x	x	x	x	x	x		x	x
		Frg 1.	301.03480					x	x	x	x	x	x	x	x	x	x	x		x	x
		Frg 2.	80.96460					x		x	x	x		x	x		x			x	x
Isorhamnetin-3-O-malonylglucoside $C_{25}H_{24}O_{15}$ ESI-	21.3 ± 0.03	Prc. Ion	563.10424	2.148	2.218	2.676	x	x	x	x	x	x	x	x	x	x	x	x		x	x
		Frg 1.	459.09270				x	x	x	x	x	x	x	x	x	x	x	x		x	x
		Frg 2.	315.05050				x	x	x	x	x	x	x	x	x	x	x	x		x	x
		Frg 3.	299.01920				x	x	x	x	x	x	x	x	x	x	x	x			
		Prc. Ion	303.04993	0.300	0.001	0.261		x	x		x	x		x	x			x		x	

Quercetin $C_{15}H_{10}O_7$ ESI+	22.4 ± 0.02	Frg 1.	201.05453								x	x									
		Frg 2.	153.01834								x	x									
		Frg 3.	165.01837								x	x									
Naringenin $C_{15}H_{12}O_5$ ESI-	23.2 ± 0.04	Prc. Ion	271.06120	0.271	0.343	0.269	x	x	x	x	x	x	x	x	x	x	x	x	x	x	x
		Frg 1.	119.04879				x	x	x	x	x	x	x	x	x	x	x	x	x	x	x
		Frg 2.	151.00226				x	x	x	x	x	x	x			x	x	x	x	x	x
		Frg 3.	107.01245				x	x	x	x	x	x	x	x	x	x	x	x	x	x	x
Luteolin $C_{15}H_{10}O_6$ ESI-	23.4 ± 0.04	Prc. Ion	285.04046	1.680	2.260	1.981	x	x	x		x	x	x	x	x	x	x	x	x	x	x
		Frg 1.	133.02834								x	x									x
		Frg 2.	151.00260								x										
		Frg 3.	175.03898								x	x									
Isorhamnetin $C_{16}H_{12}O_7$ ESI-	26.3 ± 0.08	Prc. Ion	315.05103	2.013	2.057	2.228	x	x	x	x	x	x	x	x	x	x	x	x	x	x	x
		Frg 1.	300.02685				x	x	x		x	x	x	x	x	x	x	x	x	x	x
		Frg 2.	151.00245				x	x	x		x	x	x	x	x	x	x	x	x	x	x
		Frg 3.	269.08542																		
Apigenin $C_{15}H_{10}O_5$ ESI+	26.5 ± 0.04	Prc. Ion	271.06010	-0.037	-	-	x									x			x		
		Frg 1.	153.01779				x									x			x		
		Frg 2.	119.04943				x									x			x		
		Frg 3.	145.02936																x		
Naringenin Chalcone $C_{15}H_{12}O_5$ ESI-	27.4 ± 0.06	Prc. Ion	271.06120	0.288	1.599	0.281	x	x	x	x	x	x	x	x	x	x	x	x	x	x	x
		Frg 1.	119.04879				x	x		x		x	x	x	x	x		x	x		x
		Frg 2.	151.00226					x					x						x		
		Frg 3.	107.01245							x			x	x	x	x	x	x	x		
Pinocembrin $C_{15}H_{12}O_4$ ESI-	30.6 ± 0.04	Prc. Ion	255.06628	0.241	-0.941	0.284	x	x	x	x	x	x	x	x	x	x	x	x	x	x	x
		Frg 1.	151.00241				x		x	x		x	x		x	x	x	x	x		x
		Frg 2.	213.05467				x		x	x		x	x		x	x		x	x		x
		Prc. Ion	283.06120	-	1.301	1.142								x			x	x		x	x

Biochanin A<br>$C_{16}H_{12}O_5$<br>ESI-	30.7 ± 0.03	Frg 1.	268.03634													x				
		Frg 2.	239.03364																	
		Frg 3.	168.98778																	
Glycitein<br>$C_{16}H_{12}O_5$<br>ESI+	30.7 ± 0.04	Prc. Ion	285.07575	-	-0.667	-1.526										x	x		x	x
		Frg 1.	270.05108													x	x		x	
		Frg 2.	242.05624													x				
		Frg 3.	225.05455																	
Galangin<br>$C_{15}H_{10}O_5$<br>ESI-	33.3 ± 0.03	Prc. Ion	269.04555	1.666	-	0.068	x			x		x	x			x			x		x
		Frg 1.	213.05450				x			x		x	x			x					x
		Frg 2.	223.04036																		
Glabridin<br>$C_{20}H_{20}O_4$<br>ESI-	33.7 ± 0.01	Prc. Ion	323.12888	1.317	-	-	x			x			x			x			x		
		Frg 1.	187.07590				x			x			x			x			x		
		Frg 2.	267.06570				x			x			x			x			x		
Sophoraflavanone B<br>$C_{20}H_{20}O_5$<br>ESI-	39.1 ± 0.03	Prc. Ion	339.12380	1.277	1.262	1.093	x	x	x	x	x	x	x			x	x	x	x	x	x
		Frg 1.	219.06418				x	x	x	x	x	x				x	x	x	x	x	x
		Frg 2.	119.04871				x	x	x	x	x	x				x	x	x	x	x	x
Sakuranetin +<br>Isosakuranetin<br>$C_{16}H_{14}O_5$<br>ESI-	34.4 ± 0.03	Prc. Ion	285.07685	1.980	1.873	1.844	x	x	x	x	x	x	x	x	x	x	x	x	x	x	x
		Frg 1.	119.04883				x			x	x		x			x			x	x	x
		Frg 2.	221.15330							x											
		Frg 3.	165.01802				x			x			x			x			x		
		Frg 4.	164.01011				x	x		x			x			x			x		
Piceatannol<br>$C_{14}H_{12}O_4$<br>ESI+	34.5 ± 0.06	Prc. Ion	245.08084	0.002	0.435	0.003	x	x	x	x	x	x	x	x	x	x	x	x	x	x	x
		Frg 1.	227.07080				x	x	x	x	x	x	x	x	x	x	x	x	x	x	x
		Frg 2.	215.07080									x		x	x			x			x
		Frg 3.	135.04460										x			x		x			
		Prc. Ion	371.15001	0.637	1.903	1.323	x	x	x	x	x	x				x	x	x	x	x	x
		Frg 1.	193.08650				x	x	x	x	x	x				x	x	x	x	x	x

Tetrahydrocurcumin		Frg 2.	219.06570						x								x			
$C_{21}H_{24}O_6$																					
ESI-	40.7 ± 0.04																				
Demethoxycurcumin		Prc. Ion	337.10815	1.875	1.842	1.836	x	x	x	x	x	x	x	x	x	x	x	x	x	x	x
$C_{20}H_{18}O_5$	41.8 ± 0.03	Frg 1.	119.04970				x	x	x	x	x	x	x	x	x	x	x	x	x	x	x
ESI-		Frg 2.	175.03950				x	x	x	x			x		x	x	x	x	x		x
		Frg 3.	217.05010				x	x	x	x	x	x	x	x	x	x	x	x	x	x	x
		Prc. Ion	353.13945	1.640	1.486	1.155	x	x	x	x	x	x	x			x	x	x	x	x	x
Xanthohumol		Frg 1.	207.10210				x		x	x	x	x	x			x	x	x	x	x	x
$C_{21}H_{22}O_5$	45.3 ± 0.03	Frg 2.	119.04900							x	x	x				x	x	x		x	x
ESI-		Frg 3.	145.02900				x			x	x	x				x	x	x		x	x

Table S6. Total phenolic compounds detected in *P. oceanica* tissues (target, suspected and non-target) and the maximum number observed in the plant (grey). Subclasses from left to right: cinnamic acids (CA), benzoic acids (BA), flavones (FL), flavonols (FLL), isoflavones (i-FL), flavonons/flavanones (FLN), chalcones (CHL), catechins (CT), prenylated isoflavonoids (pr-IsF), dihydroflavonols (d-FLL), curcuminoids (CU) and stilbenes (ST).

		CA	BA	FL	FLL	i-FL	FLN	CHL	CT	pr-IsF	d-FLL	CU	ST
**EE3**	Leaf	7	2	2	7	1	4	2	2	1	1	2	1
	Rhizome	3	2	3	7	1	5	2	3	0	1	2	1
	Root	3	2	3	8	1	5	2	2	0	1	2	1
**FAN7**	Leaf	7	2	3	5	1	5	2	2	1	0	2	1
	Rhizome	3	2	3	7	3	5	2	3	0	1	2	1
	Root	2	2	2	7	2	5	2	2	0	0	2	1
**AL2x**	Leaf	7	2	4	6	1	4	2	2	1	1	2	1
	Rhizome	3	2	3	7	1	4	2	3	0	1	2	1
	Root	3	2	3	7	1	4	2	3	0	1	2	1
**AL3**	Leaf	7	2	3	7	1	5	2	2	1	1	1	1
	Rhizome	3	2	3	6	3	4	1	3	0	1	1	1
	Root	3	2	3	6	1	4	1	3	0	1	1	1
**CG4**	Leaf	6	2	4	7	1	5	2	2	1	1	2	1
	Rhizome	3	2	3	6	3	5	2	3	0	1	2	1
	Root	3	2	3	7	3	5	2	3	0	1	2	1
Max.		7	2	4	8	3	5	2	3	1	1	2	1
Average	Leaf	6.8	2.0	3.2	6.4	1.0	4.6	2.0	2.0	1.0	0.8	1.8	1.0
	Rhizome	3.0	2.0	3.0	6.6	2.2	4.6	1.8	3.0	0.0	1.0	1.8	1.0
	Root	2.8	2.0	2.8	7.0	1.6	4.6	1.8	2.6	0.0	0.8	1.8	1.0
